# Reimagining primary health care: a historical and contemporary scoping review of community-based primary health care models and innovations

**DOI:** 10.1016/j.pmedr.2026.103390

**Published:** 2026-01-27

**Authors:** Sanjaya Acharya, Shiva Raj Mishra, Lorenz von Seidlein, Bipin Adhikari, Daniel M. Parker

**Affiliations:** a Academy for Data Science and Global Health, Kathmandu, Nepal; b School of Medicine, University of Western Sydney, Sydney, Australia; c Mahidol-Oxford Tropical Medicine Research Unit, Faculty of Tropical Medicine, Mahidol University, Bangkok, Thailand; d Centre for Tropical Medicine and Global Health, Nuffield Department of Medicine, University of Oxford, Oxford, United Kingdom; e Joe C Wen School of Population and Public Health; University of California, Irvine, USA

**Keywords:** Community-based primary health care, Community health workers, Primary health care, Health equity, Digital health, Global health

## Abstract

**Objectives:**

Community-based primary health care (CBPHC) has long underpinned health service delivery in resource-limited settings. However, demographic shifts, increasing chronic disease burdens, and digital transformations challenge its sustainability. This review synthesizes historical and contemporary evidence on CBPHC to assess effectiveness, identify limitations, and outline future directions toward universal health coverage (UHC).

**Methods:**

Using the Arksey and O'Malley framework, we conducted a scoping review of global literature from 1975 to 2025 across PubMed, Scopus, Web of Science, Google Scholar, and grey sources. Data were thematically analyzed into categories capturing evolution, achievements, challenges, and future directions.

**Results:**

A total of 134 documents were reviewed. CBPHC improved access to essential services, particularly maternal and child health, infectious disease control, and health promotion. Programs led by community health workers and volunteers strengthened systems but faced persistent barriers such as attrition, limited funding, and weak integration. Case studies from Nepal, Ethiopia, Brazil, and Rwanda showed improved maternal and child outcomes and pandemic preparedness and resilience. Emerging challenges include syndemics, demographic shifts, and urbanization.

**Conclusions:**

CBPHC remains vital for advancing universal health coverage. Its sustainability depends on evolving into a diagonally integrated, people-centered, and digitally enabled model supported by equitable investment in governance, workforce training, and community engagement.

## Highlights

• Community-based primary health care strengthens equity through locally responsive care.
• Community-based primary health care faces ongoing funding and integration challenges.
• Urgent investment is needed in digital innovation, governance, workforce, and communities.

## 1 Introduction

Community-based primary health care (CBPHC) delivers essential health services in underserved areas through trained local providers (Perry et al., 2014). Emphasizing access, affordability, and cultural relevance, CBPHC bridges gaps where formal systems are limited or overstretched and has deep historical roots (S1-S3). CBPHC systems have deep historical roots, including, China's Barefoot Doctors (1960s), Nepal's Female Community Health Volunteers (FCHVs) (1988) (Wen, 1975; Panday et al., 2017; Musoke et al., 2021). Earlier models such as Russia's feldshers exemplified localized, people-centered care (S4). These historical models evolved pragmatically pressed by the shortages of trained physicians or institutions (Van Lerberghe, 2008; Tollman, 1991).

CBPHC has driven global health improvements by extending services to remote areas, building trust in health systems (Black et al., 2017). These programs extend services to remote areas, build trust in health systems, and promote community ownership (S5, S6). Beyond service delivery, CBPHC functions as a cornerstone of public health, by promoting disease prevention, and more equitable access to essential health services (S7).

CBPHC adaptation is crucial as urbanization, climate change, demographic shifts, and rising chronic diseases reshape the global health landscape (Damte et al., 2023; Monaco et al., 2020). The COVID-19 pandemic exposed health system inequalities worldwide, reinforcing the need for strong, community-rooted healthcare models centered on CHWs (Tran et al., 2020).

Community health workers are the conduit between communities and the health system. While analyses of South Asian CHW programs highlight their roles, broader systemic transitions remain overlooked. Positioning CHWs within CBPHC's global evolution is vital amid demographic and technological change (Shrestha et al., 2024). This paper reexamines community-based primary health care in a changing global health scenario, including the influence of digital technologies such as AI. We review historical and contemporary CBPHC models to summarize their effectiveness and limitations, identify emerging challenges, and outline future directions to align with UHC goals.

## 2 Methods

### 2.1 Review design

**Fig. 1:**
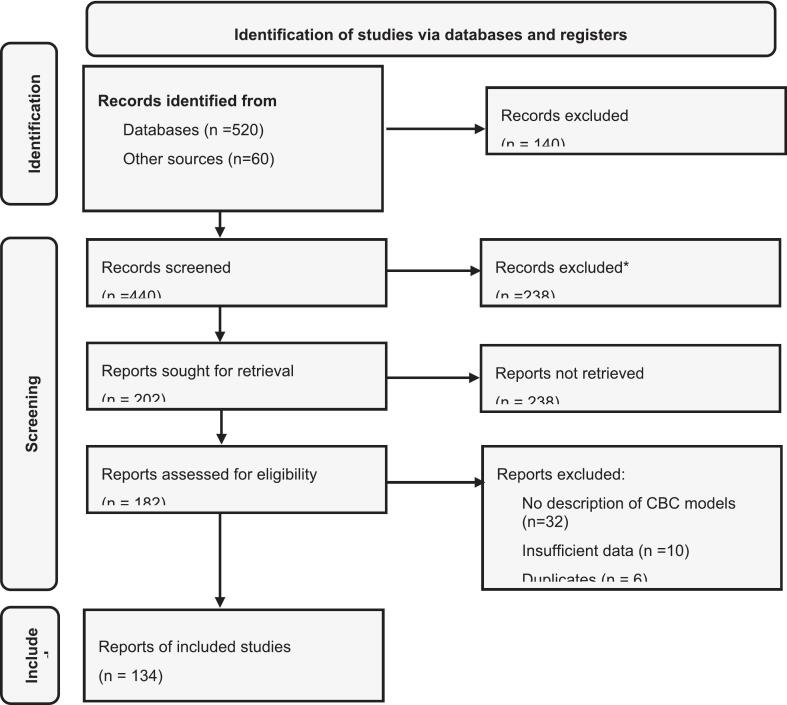
PRISMA flow diagram illustrating study identification, screening, eligibility assessment, and inclusion for the scoping review of community-based primary health care literature, 1975–2025.Search strategy and inclusion criteria.

This scoping review utilized the methodological framework proposed by Arksey and O'Malley. The review followed five stages: (Perry et al., 2014) refining research questions through discussions; (Wen, 1975) identifying relevant studies; (Panday et al., 2017) searching major databases and grey literature; (Musoke et al., 2021) collating and synthesizing data; and (Van Lerberghe, 2008) discussing findings with experts to assess relevance of results (Arksey and O'malley, 2005; Levac et al., 2010). These steps guided the key themes discussed in subsequent sections (Fig. 1).

A comprehensive search across PubMed, Scopus, Web of Science, and Google Scholar was conducted retrieving 520 documents including 60 grey literatures for example from institutional websites, such as WHO, UNICEF, the World Bank, and national health ministries.

The search strategy used various combinations of terms including: “Community-based care” OR “community health services” OR “community-oriented primary care” OR “community health worker” OR “CHW” OR “volunteer health worker” OR “digital health” OR “telemedicine” OR “mobile health” OR “mHealth” OR “artificial intelligence” OR “AI in health care” OR “machine learning” OR “health system integration” OR “diagonal integration” OR “horizontal integration” OR “syndemic” OR “syndemics” OR “pandemic preparedness” OR “COVID-19” OR “primary health care” OR “universal health coverage” OR “low-and-middle income countries” OR “LMICs”.

We reviewed all the collated reports, retaining a total of 134 documents. Literature meeting the inclusion criteria included: (i) community-based care models; (ii) emerging issues such as digital health and AI integration; or (iii) primary and community health systems were retained. Non-English publications and conference abstracts were excluded.

Articles were excluded if focused on hospital-based care, lacked sufficient detail or relevance to CBPHC or CHW implementation, or omitted content on syndemics (co-occurring diseases that interact biologically and socially), integration, or digital (AI) applications.

### 2.2 Data abstraction

Data were extracted to collate the details on author, year, methods, findings, limitations, and future directions. SA extracted data into tables and prepared narrative summaries, with input from all authors. Literature was organized into five themes: defining CBPHC, its evolution and impact, barriers and facilitators, and digital or AI-enhanced delivery. Aligning with the scoping review standards, studies were not discriminated based on the quality but on the relevance.

### 2.3 Synthesis and organization of findings

Studies underwent iterative thematic synthesis, refining predefined themes and generating inductive subthemes to capture recurring and context-specific insights. Findings were summarized in tables to identify overlaps and gaps, then consolidated and presented across major themes based on relevance and significance (Table 1**)**. Additional historical, contextual, and technical sources are provided in the supplementary references (S1-S40).

**Table 1:** Thematic synthesis and organization of findings from community-based primary health care studies evaluating health outcomes across low- and middle-income countries.

Themes	Topics
Defining community-based primary health care.	Scope and definitions, CHWs, volunteers, peer-support models, role in UHC
Evolution and impacts of community-based primary health care.	Historical trajectories, integration into PHC, improvements in MCH, infectious disease control, case studies (Ethiopia, Nepal, Brazil)
Emerging challenges and changing landscape.	Syndemics (HIV-TB, COVID-19–NCDs), demographic transitions, urbanization, weakening social structures
Digital disruption: AI and technology in community care.	mHealth, telemedicine, AI decision-support tools, equity and governance issues
Future directions and policy implications.	Diagonal integration, sustainable financing, CHW training, digital readiness, community engagement

## 3 Results

### 3.1 Characteristics of included studies

Of the 134 documents included in this review (1975–2025), the majority were narrative reviews, program descriptions, or policy commentaries (62%), followed by systematic/scoping reviews and meta-analyses (22%), and primary empirical studies (16%) comprising qualitative research, cross-sectional surveys, mixed-methods designs, and two randomized trials. The cutoff year was selected to align with the 1978 Alma-Ata Declaration on primary health care. Most studies were country-specific (71%), with the remainder global or multi-country. Publications increased after 2000, peaking in the 2010s–2020s amid growing focus on UHC, NCDs, digital health, and pandemic preparedness.

### 3.2 Defining community-based primary health care

Included articles covered diverse CBPHC contexts, including policy development, implementation strategies, and applications. Most described CBPHC as an evolving response to gaps in traditional healthcare systems, defining it as a holistic, community- and home-based approach delivered largely by non-physician or lay health providers (Bhattarai et al., 2020). Historically CBPHC has been planned for rural populations, extending its utility to emerging and re-emerging conditions. CBPHC also draws from social movements like the Black Panther Party, which in the 1960s–70s advanced community-led health programs addressing medical and social determinants (S8).

CBPHC should incorporate strong community engagement to improve service access and uptake. Key actors, including community health workers, youth volunteers, and peer supporters, operate under technical supervision and provide services ranging from basic malaria diagnosis to management of tuberculosis and chronic diseases. The effectiveness of community health workers depends on strong primary care systems, supportive policies, intersectoral collaboration, efficient referrals, and continuous monitoring. These components of CBPHC are vital in strengthening the health system and achieving universal health coverage (UHC). Achieving UHC requires equitable access to essential services alongside financial protection for healthcare costs. Some CBPHC models are led by community health workers or volunteers offering home-based midwifery for pregnant women and peer support approaches for HIV and TB (Shahmanesh et al., 2021).

Community health workers are trusted frontline health workers with intimate knowledge of their communities and are best positioned to bridge linguistic and cultural gaps, promoting health education, care coordination, resource linkages, and patient self-efficacy. Some of their roles entailed health education, advocacy, outreach, monitoring, and data maintenance. Volunteer-led CBPHC models, especially in rural settings rely on trained, unpaid community members who complement formal health systems by delivering preventive, promotive, curative, and psychosocial support services. These volunteers, often as familiar community members, provide essential home-based care, particularly in areas with limited access to formal health services. In peer-support models, volunteers with lived experience (e.g., mental illness, HIV, or addiction) assist others facing similar challenges, fostering empathy, reducing stigma, and building social support. Integrated within the primary health care systems, volunteers work side by side with formal health workers in clinics or community outreach in screening, monitoring and referring of patients (Panday et al., 2017; Safary et al., 2021).

### 3.3 Evolution and impact of community-based primary health care

**Fig. 2:**
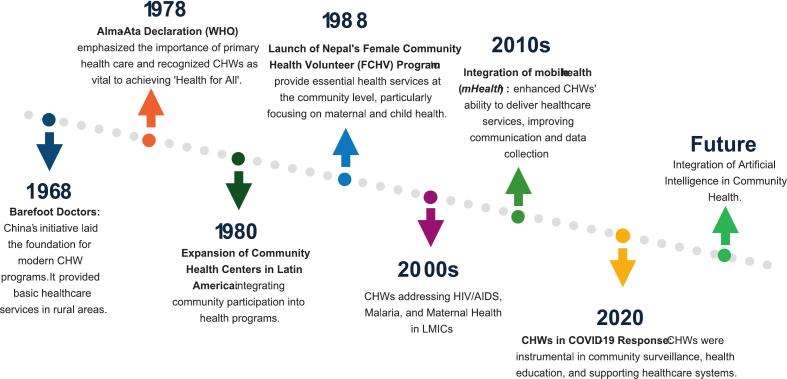
Historical timeline showing the evolution of community health worker- led and community-based primary health care programs globally, from pre-20th century informal care models to contemporary integrated systems.

Rooted in traditions such as China's “barefoot doctors,” Russia's “feldshers,” and indigenous practices, CBPHC has evolved from informal care to structured public health programs (Tollman, 1991; Rössler et al., 1994; Daniş, 2008). These initiatives reflect the use of community-based health workers as a cornerstone of primary care, a role reaffirmed by a compendium of 29 case studies from national CHW programs across diverse low- and middle-income countries (S9-S11). The 1978 Alma-Ata Declaration reinforced this, emphasizing UHC through strengthened community-oriented primary healthcare. Over the past century, CBPHC has expanded healthcare access and improved population health through services such as immunization, maternal and childcare, disease control, and health education (Fig. 2).

Before the 20th century, informal caregivers, such as traditional birth attendants, healers, and community elders provided early forms of CBPHC across indigenous and rural societies (Aziato and Omenyo, 2018). These caregivers drew on indigenous, spiritual, and ecological knowledge, using local resources and holistic approaches (S12–14). Their community-centered practices relied on trust, social cohesion, and lived experience rather than formal training.

For instance, China's Barefoot Doctors program trained rural villagers for 3–6 months in basic care and health education during the Cultural Revolution (1966–1976), significantly expanding healthcare access and improving outcomes in underserved communities (Wen, 1975). Often farmers, these “barefoot doctors” received 3–6 months of training in Western and traditional Chinese medicine, hygiene, and reproductive health, providing essential care after fieldwork hours. Their efforts led to major public health gains, reducing infant mortality from 200 to 34 per 1000 live births and raising life expectancy from 35 to 60 years (1962–1982). These early initiatives laid the foundation for modern primary healthcare, emphasizing local capacity building, community trust, and cultural competence (Perry et al., 2014).

Inspired by this success and the Alma-Ata Declaration, many countries adopted CBPHC models such as Nepal's Female Community Health Volunteers, Uganda's Village Health Teams, and Brazil's Community Health Agents, all contributing to improved community health outcomes (Panday et al., 2017; Musoke et al., 2021; Macinko and Harris, 2015) (Table 2).

**Table 2:** List of various community-based primary health care programs, their year of establishment, objectives, and roles in service delivery across countries and regions.

Community based primary health care program.	Country	Established (year)	Mission	Role
Feldsher System	Russia	1870	Deliver basic medical services in rural areas through trained non-physician practitioners.	Feldshers provided preventive, diagnostic, and basic curative services, especially where physicians were unavailable.
Ujamaa Villages	Tanzania	1967	Promote collective farming and equitable access to social services through villagization.	Implemented communal living and farming, with integrated health and education services in rural villages.
Barefoot Doctors Program	China	1968	Provide basic healthcare services to rural populations lacking access to trained physicians.	Trained rural individuals to deliver primary care, health education, and preventive services in their communities.
Bangladesh Rural Advancement Committee (BRAC's) *Shasthya Shebika* Program	Bangladesh	1970	Empower women to deliver essential health services in rural areas.	Female community health workers provide health education, maternal and child health services, and referrals.
Jamkhed Comprehensive Rural Health Project (CRHP)	India	1970	Improve rural health through community participation and empowerment.	Community-led development programs including health education, women's groups, and sanitation initiatives.
Aboriginal Medical Service (AMS) Redfern	Australia	1971	Deliver culturally appropriate health services to Aboriginal communities.	First Aboriginal community-controlled health service provides culturally appropriate, community-controlled healthcare and support services to improve the health and wellbeing of Aboriginal and Torres Strait Islander people; model replicated nationwide.
Female Community Health Volunteers (FCHVs)	Nepal	1988	Deliver primary health care to women and families in rural districts.	Volunteers provide health education, distribute vitamin A, and manage childhood pneumonia.
Lady Health Workers Program	Pakistan	1994	Provide primary health care and family planning services at the doorstep.	Trained female workers deliver preventive, promotive, and basic curative care in rural communities.
Comprehensive Community Based Rehabilitation in Tanzania (CCBRT)	Tanzania	1994	Provide disability and rehabilitation services to low-income Tanzanians.	Offers ophthalmology, orthopaedics, maternal health, and rehabilitation services.
Uganda's Village Health Teams (VHTs)	Uganda	2001	promote community participation in the delivery of essential health services and empower households to take responsibility for their own health.	volunteers of health promoters and community mobilizers, health education, promote preventive practices, and conduct home visits.
Ethiopia's Health Extension Workers	Ethiopia	2003	expand access to essential preventive, promotive, and basic curative health services at the community level	Provide health services under four areas: hygiene and environmental sanitation, disease prevention and control, family health services, and health education and communication.
Wellbody Alliance	Sierra Leone	2006	Ensure healthcare as a human right in Kono District.	Operates primary care facilities, women's centre, and provides HIV/AIDS and TB treatment.
National Community Health Assistant Program	Liberia	2016	Extend health services to remote communities beyond 5 km from health facilities.	Paid community health assistants provide integrated primary health services.

Governments increasingly recognized the value of traditional healers, integrating them into national health systems through trained birth attendants and practitioners of Ayurveda, Unani, Homeopathy, and related disciplines. Scholars increasingly advocate integrating traditional medicine into formal healthcare, particularly within CBPHC-based primary care, despite advancements in modern health systems (Lyngdoh, 2022). Over time, adapted CBPHC models have promoted community ownership and participation by integrating traditional medicine into primary care, ensuring locally based providers can enhance access and engagement (S15). Despite growing support for integrating traditional medicine into CBPHC, resistance persists in many high-income countries where strict medical regulation and evidence-based funding restrict diagnosis and treatment to licensed biomedical professionals due to safety concerns (Ijaz and Boon, 2018) (S16). In Australia, debates persist over Aboriginal healers' roles amid professional and institutional resistance (S17, S18). These tensions highlight the challenge of balancing cultural inclusion and regulation.

**Fig. 3:**
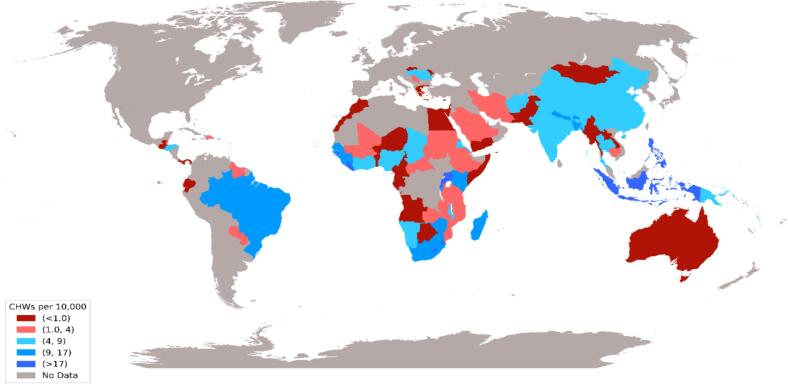
Distribution and density of community health workers per 10,000 population across countries with established community health worker programs, based on latest World Bank data. (https://data.worldbank.org/indicator/SH.MED.CMHW.P3).

The evolution of CBPHC has driven major public health achievements, advancing UHC and addressing key social determinants of health. CBPHC provides cost-effective care and supports outbreak response (S18–20). In Vietnam, Village Health Workers effectively prevented and managed hypertension and diabetes through community awareness and local interventions (S21). World Bank data show extensive but heterogeneous CHW networks in sub-Saharan Africa and Southeast Asia, reflecting global variation in their integration within national health systems (Fig. 3).

A major achievement is expanded healthcare access in remote areas through trained CHWs. Ethiopia's 2003 Health Extension Program advanced prevention, family health, hygiene, and health promotion in rural communities. This contributed to significant reduction in one of their health indicators—under five mortalities from 203 per 1000 live births in 1990 to 58 in 2016. In India, the 2006 Accredited Social Health Activist (ASHA) program improved maternal care, increasing first ANC visits by 17%, skilled birth attendance by 26%, and facility-based deliveries by 28% in rural communities. In Bangladesh, BRAC's community health volunteers promoted oral rehydration therapy, significantly reducing child deaths from diarrheal disease between the 1980s and early 2000s (Victora et al., 2000). CBPHC has improved child nutrition, maternal health, and expanded access to family planning and immunization (supplementary Fig. 4) (Khatri et al., 2017).

Through participation and local leadership, CBPHC has strengthened community ownership and accountability. In Ethiopia, the Health Extension Program trained female health workers and engaged communities in setting priorities and supporting services, improving outcomes such as immunization coverage and maternal health CBPHC's evolution varies globally, yet integration into formal systems consistently enhances effectiveness (Table 3).

**Table 3:** Evolution and population-level health impacts of community-based primary health care models in Rwanda, Papua New Guinea, and Moldova.

Country	Community based primary health care evolution and its impact
Rwanda	Rwanda had the lowest life expectancy of any country globally from 1989 to 1997, primarily due to burden of communicable diseases and unsafe birth practices, further exacerbated by the 1994 genocide, and health workforce setback further plagued the health system of the country. However, Rwanda's health system now is often cited as successful example of CBC fostering community ownership, accountability and cost effectiveness in resource-limited settings as CHWs are elected by their communities and trained to provide primary health services and health education. It is observed that Rwanda's CBC strategy contributed to 70% decline in under-five mortality between 2002 and 2011, demonstrating the impact of this model.
Papua New Guinea	Papua New Guinea (PNG) struggled with poor health indicators, with life expectancy around 55 years in early 2000s due to infectious diseases like malaria and tuberculosis, maternal health issues, and geographic isolation limiting healthcare access. The health workforce density was below 0.3 health workers per 1000 population. PNG's community health workers, chosen by local communities and trained to provide primary care and health promotion, have played a key role in bridging healthcare gaps. These community-based approaches contributed to a decline in under-five mortality from about 100 per 1000 live births in 1990 to approximately 57 per 1000 in 2015, emphasizing their importance in resource-constrained, hard-to-reach settings.
Moldova	Moldova faced health system challenges post-Soviet Union dissolution, with life expectancy dropping to around 66 years in the 1990s, partly due to rising communicable diseases such as tuberculosis and HIV, poor maternal health services, and economic hardships. Workforce shortages and infrastructure weaknesses (approximately 2 physicians per 1000 population) strained healthcare delivery. The country introduced community health initiatives involving local health promoters, which improved early disease detection and maternal-child health services. This community-centered approach contributed to a steady increase in life expectancy to over 70 years by 2015 and a decline in maternal mortality from 56 to 22 deaths per 100,000 live births over the same period, despite limited resources.

CBPHC structures have shown resilience during health crises. During the 2014–2016 Ebola outbreak, Sierra Leone mobilized CHWs for surveillance, contact tracing, and health education, ensuring early detection and continuity of immunization services. During COVID-19, Vietnam's village health workers supported early detection, contact tracing, and quarantine enforcement, helping contain outbreaks and prevent widespread transmission during the first 2020 wave (Tran et al., 2020).

Despite successes, CBPHC faces major challenges, notably inconsistent training and supervision leading to variable clinical competence and heterogeneity in quality of care. In resource-limited settings, task shifting and sharing help address workforce gaps, but CHWs often face excessive workloads and limited support, reducing care quality and contributing to burnout and attrition (Pandya et al., 2022). Volunteer attrition is common due to low compensation, limited training, and quality concerns. In Tanzania's Simiyu Region, 12.7% of CHWs left within four years, citing poor financial incentives, work demands, family pressure, and relocation (S22). In western Kenya, 33% of CHWs in HIV home-based care left programs for similar reasons These patterns highlight sustainability and cost-efficiency concerns, as frequent CHW turnover increases recruitment and training expenses. Yet, financial constraints hinder fair compensation, even though underfunding ultimately drives higher costs through attrition and inefficiency (S23). CBPHC programs often fragment due to poor coordination, causing duplication and inefficiency across governments, NGOs, and donors (Barr et al., 2019). The 2019 World Health Assembly highlighted uneven CHW integration, linked to weak inclusion in health system planning, coordination, and partnerships. Uganda's 2001 CHW program addressed workforce shortages and disease burdens but also revealed donor-driven fragmentation, with overlapping initiatives in maternal and communicable diseases causing parallel reporting, conflicting priorities, and resource inefficiencies (Afzal et al., 2021).

Integrating CBPHC into formal systems is hindered by weak coordination and policy gaps. Traditional healers and informal providers, though vital in rural care, remain excluded, causing fragmented services and missed opportunities for synergies.

CBPHC programs often lack strong monitoring systems, limiting evaluation and scale-up. In Uganda's Wakiso District, only 20% of CHWs performed well; refresher training improved outcomes, but frequent stock-outs persisted. Cultural barriers, provider mistrust, and gender norms limit CHW access and program reach (Sieverding and Beyeler, 2016).

Sustainable funding remains limited, with CBPHC programs relying on short-term donor support (Sieverding and Beyeler, 2016). These challenges highlight the need for strategic planning, integration, and sustained policy commitment to strengthen CBPHC through investment, infrastructure, and alignment with formal health systems.

### 3.4 Emerging challenges and a changing landscape

Global health is rapidly transforming, with emerging challenges in access and delivery. Syndemics, such as HIV and tuberculosis co-infection, illustrate how interacting conditions worsen disease outcomes (Chaisson and Martinson, 2008). During COVID-19, individuals with chronic conditions faced higher risks of severe disease and outcomes. These dual burdens strained fragile health systems, with higher mortality and severity among affected individuals in resource-limited settings (Nachega et al., 2021). Integrated, decentralized CBPHC models can bundle services—combining HIV, TB, malaria, or COVID testing with community engagement, to improve diagnosis and outcomes.

Demographic transitions, such as shifts in fertility, mortality, and aging are reshaping population structures and disease patterns. As infectious diseases give way to chronic NCDs, health systems face additional pressures. A key challenge is the mismatch between resource allocation for acute care and the growing need to manage chronic conditions. These shifts pose challenges and opportunities for CBPHC delivery. Elderly and disabled individuals often face barriers to accessing care.

CBPHC strategies such as home-based care, CHW-led screening, education, and social support can serve vulnerable groups. In LMICs, overlapping infectious and chronic disease burdens intensify health challenges and strain already limited systems. Demographic transitions also create youth bulges—large populations aged 15–30—driven by declining fertility rates (S24, S25). Large youth populations face challenges, including early childbearing and sexually transmitted infection mental health concerns, substance abuse, unemployment related stress.

Rapid urbanization without adequate infrastructure heightens health risks through overcrowding, pollution, and increased infectious diseases and NCDs. Urban slums' limited healthcare access causes delayed diagnosis, poor treatment, and worsened health inequalities among the urban poor (Park et al., 2022). Addressing these issues demands inclusive urban health strategies integrating CBPHC, strengthening primary care, and engaging communities, especially migrants, informal workers, and slum populations (Shafique et al., 2024).

Social structures are also shifting, with rural areas rapidly transitioning toward urbanization (S26). Traditional family and community networks have long shaped caregiving, health, and social support roles (S27). Gender roles, often viewed as fixed, have varied across time and place. Contemporary shifts both challenge and reinforce traditional norms, yet history shows these roles are socially constructed and fluid, shaped by economic, political, and cultural contexts (S28). Globalization and migration have reshaped social structures, fragmented families and leaving elders, women, and children without consistent care or support (S29). Migrants face legal, cultural, and language barriers to care, increasing risks of mental illness and disease.

In rapidly urbanizing and high-income settings, CBPHC faces challenges from shifting social norms. Communal living has transitioned to nuclear or individual households; in Nepal, about 70% of families now live in nuclear arrangements (Rai, 2023). This transformation is nonlinear; social structures evolve into different dimensions, for instance, some kin forms fade, others adapt under cultural, economic, and political forces. This transition erodes community cohesion and informal support vital to CBPHC. In many LMICs, strong social bonds sustain CHWs, whereas urban privacy, independence, and fragmented living weaken volunteerism and neighborhood solidarity (S30). This shift weakens informal care such as CBPHC, increasing reliance on institutional care; as more women join the workforce, reducing traditional caregiving roles (Moen et al., 1994) (S31, S32).

Evolving social structures are reshaping relationships and healthcare access, heightening vulnerability among migrants, women, elders, and youth. Responsive, people-centered systems must strengthen CBPHC to offset declining traditional support networks. Health services must be culturally sensitive, address diverse households, and tackle isolation, mental health, and gender disparities.

### 3.5 Digital disruption: AI and technology in community care

Digital health, AI, and mobile tools are reshaping primary care in LMICs, improving access and efficiency while redefining the notion of ‘community’ in healthcare delivery. Growing use of digital health reduces reliance on clinics, while online health-seeking behaviors often foster misinformation, disinformation, and risky self-diagnosis (Melchior and Oliveira, 2022). ‘Community’ in healthcare now extends beyond geography to digitally connected networks. The COVID-19 pandemic accelerated adoption of digital tools such as telemedicine and virtual consultations amid lockdowns and social distancing.

Digital health enables remote care through telemedicine, mobile health apps, and virtual consultations (S33). Virtual models offer medical advice and treatment monitoring without clinic visits, reducing travel costs, improving continuity, and ensuring timely care for rural or low-income populations (S34).

AI is transforming healthcare, improving workforce efficiency and patient outcomes, for instance, supporting screening and diagnosis through machine and deep learning, analyzing medical images, lab results, and records efficiently. It enables early detection of diseases such as cancer and diabetic retinopathy, identifying anomalies often missed by clinicians (S35). AI-driven decision support aids clinicians with risk assessment, diagnosis, and evidence-based treatment recommendations (Supplementary Table 4) (S36). CHWs can use AI tools to expand outreach and close diagnostic gaps. Generative AI for instance, can generate original outputs, including text, images, audiovisual content, and code, based on previously learned data (S37). Growing interest in generative AI and large language models offers promise but, without safeguards, risks reinforcing existing healthcare inequalities through biased data. Biases may arise from underrepresentation of populations, for instance, it can spontaneously replicate and normalize the neglect of minorities and vulnerable populations, including their needs. Algorithmic bias can embed systemic inequities as scientific truth, perpetuating malpractice in diagnosis, treatment decisions, and resource allocation. AI systems may exhibit cultural or linguistic insensitivity, failing to accommodate non-dominant languages and norms (S38). Overreliance on AI can overlook human judgment, while limited transparency and accountability leave users uncertain about how recommendations are generated (S39). Preventing harm requires diverse training data, culturally inclusive design, and human oversight to validate AI-driven health decisions. It raises concerns about equity, digital literacy, overreliance on technology, and depersonalized care.

Challenges include poor connectivity, logistics gaps, bias, ethics, and privacy. New technologies redefine ‘community,’ shifting it from physical spaces to digital networks. Without grounding in local culture, technology risks dehumanizing care and reducing community acceptance. Digital technologies are best positioned to complement rather than replace human capacity in healthcare services (S40).

## 4 Discussions

This review tracks historical developments in community-based programs, as well as barriers and facilitators in an age of rapid technological advancement and AI. Policy makers must prioritize integrating CBPHC into national health strategies, supported by sustainable financing and intersectoral collaboration. Strengthening the training of CHWs is essential when there is a need of multi-skilled, digitally literate healthcare workers capable of delivering integrated health services in the community. Investment in community level infrastructure, including reliable digital tools, mobile connectivity, and supply chain systems will be pivotal in efficient and data-driven care.

Community engagement enhances CBPHC's accountability. It can ensure that the CBPHC's care is acceptable/culturally tailored to the community through feedback cycles, dialogue, and even through identification of priority areas. Community engagement also enhances the access and acceptability through wide reach, engagement occurring at informal social spaces (family, peers, and community members even when not deliberated). Community engagement can become the conduit for health promotion (healthy behavior, vaccination, improvement of water and sanitation), disease prevention and thus can be an extended arm of CBPHC.

The sustainability of CBPHC increasingly requires effective multi-sectoral action, as many key drivers of health lie outside the formal health sector, including climate change, urbanization, and social protection systems. The current global health scenario is characterized by declining development assistance, and political shifts that have weakened support for equity-oriented health initiatives. These trends threaten the continuity of community focused programs in LMICs, which depend on stable financing and cross-sectoral coordination. In this context, CBPHC and CHW platforms function not only as service delivery mechanisms but also as local interfaces for integrated preventive action across sectors. Strengthening these linkages will be critical for sustaining preventive gains and building resilient health systems amid growing global uncertainty. These broader structural shifts provide important context for understanding why CBPHC must adapt and innovate.

While mHealth and digital platforms have already shown promise, emerging AI systems offer the potential to further enhance efficiency, decision-making, and personalization of care. AI-powered tools can support CHWs in screening, triage, and referral, especially in underserved areas. These tools can help standardize protocols, reduce delays in diagnosis, and enable real-time data-driven responses. AI can also facilitate natural language processing to translate or tailor health messages into culturally appropriate content, improving health literacy and community engagement. However, to ensure ethical and equitable deployment, AI integration must be guided by robust governance frameworks, data privacy protections, and inclusive design processes that reflect the realities of low-resource and diverse community contexts.

The scoping review enabled the inclusion of a wide range of literature, capturing diverse perspectives on CBPHC including its historical evolution, links to primary health care, and real-world implementation. Expert consultation enhanced the relevance of the review, drawing on their experiential and epistemic insights. Notably, experts emphasized key themes such as sustainability, financing, dependency, and integration into health systems as central to CBPHC.

The review may reflect author bias due to prior experience in community-based health services, research, and implementation. Additionally, the heterogeneity of findings (e.g., impacts) may have been reduced by the authors' *a priori* assumptions related to community-based primary health care. The absence of systematic quality appraisal limited quantitative impact assessment, and lack of meta-analysis are further limitations.

Recognizing the complexity of the topic, this review aimed to contextualize findings for real-world application. It offers a consolidated account of CBPHC's development, core characteristics, and implications for future directions.

## 5 Conclusions

Community-based primary health care plays a vital role in strengthening primary health care and advancing population health, particularly in underserved settings. This review shows that CBPHC improves access to essential health services, supports prevention and health promotion, and promotes more equitable health outcomes by addressing community-level needs.

However, its impact is often constrained by workforce attrition, fragmented implementation, and declining funding. Strengthening CBPHC requires greater integration within national primary health care systems, supported by sustained investment and effective governance. Digital innovations may enhance service delivery and coordination, but their impact depends on ethical and context-sensitive implementation. Overall, CBPHC represents a core preventive strategy for building resilient and people-centered health system.

## CRediT authorship contribution statement

**Sanjaya Acharya:** Writing – review & editing, Writing – original draft, Visualization, Software, Resources, Project administration, Methodology, Investigation, Formal analysis, Data curation, Conceptualization. **Shiva Raj Mishra:** Writing – review & editing, Writing – original draft, Visualization, Validation, Supervision, Software, Resources, Project administration, Methodology, Investigation, Formal analysis, Data curation, Conceptualization. **Lorenz von Seidlein:** Writing – review & editing, Validation, Supervision, Methodology, Conceptualization. **Bipin Adhikari:** Writing – review & editing, Writing – original draft, Visualization, Validation, Supervision, Resources, Project administration, Methodology, Investigation, Conceptualization. **Daniel M. Parker:** Writing – review & editing, Writing – original draft, Validation, Supervision, Resources, Methodology, Conceptualization.

## Funding

The authors declare that there was no specific funding for the study. LvS and BA are affiliated with the Mahidol Oxford Tropical Medicine Research Unit (MORU), which is funded by the Wellcome Trust, UK (220211/Z/20/Z). For the purpose of Open Access, the author has applied a CC BY public copyright license to any Author-Accepted Manuscript version arising from this submission. The funder had no role in the writing or preparation of the manuscript.

## Declaration of competing interest

The authors declare that they have no known competing financial interests or personal relationships that could have appeared to influence the work reported in this paper.

## Data Availability

All data used in this study are available online, as described in the manuscript

## Appendix A Supplementary data

Supplementary material 1 Image 1

Supplementary material 2 Image 2

Supplementary references Image 3
