# Supplementary material for: Reimagining primary health care: a historical and contemporary scoping review of community-based primary health care models and innovations
Source: Prev Med Rep. 2026 Jan 27;62:103390. doi: 10.1016/j.pmedr.2026.103390 (PMC12877820; doi:10.1016/j.pmedr.2026.103390)
Supplement: Supplementary file 1 — Supplementary material 1 [file mmc1.docx]

**Nepal**

**Female Community Health Volunteer Program**


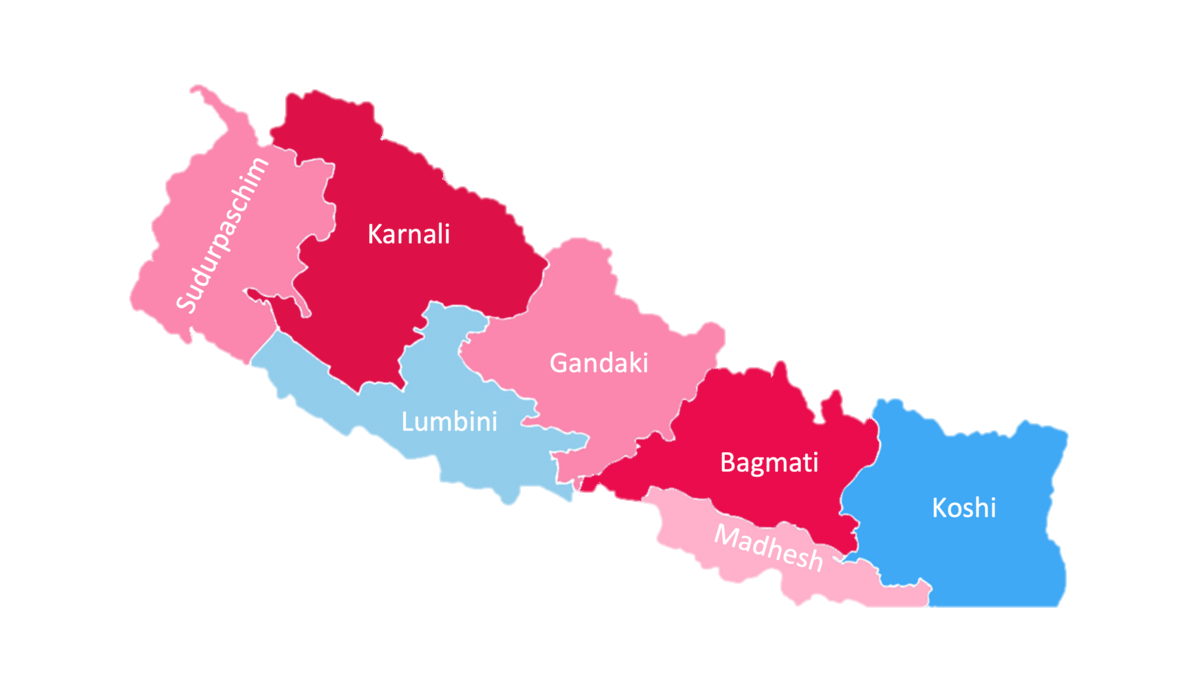


**Overview:**

Female Community Health Volunteer Program established in 1988, is a cornerstone of the country’s community-based primary health care system.

**Key Features:**

- Volunteer-based model with over 50,000 female CHWs, typically selected by their communities.
- Focuses on maternal and child health, nutrition, immunization, and family planning.
- Basic training and regular refresher courses, with strong supervision from health posts.

**Impact:**

- achievements in reducing maternal and under-five mortality.
- health promotion for remote populations.
- critical role during the COVID-19 response.

**Ethiopia**

**Health Extension Program**


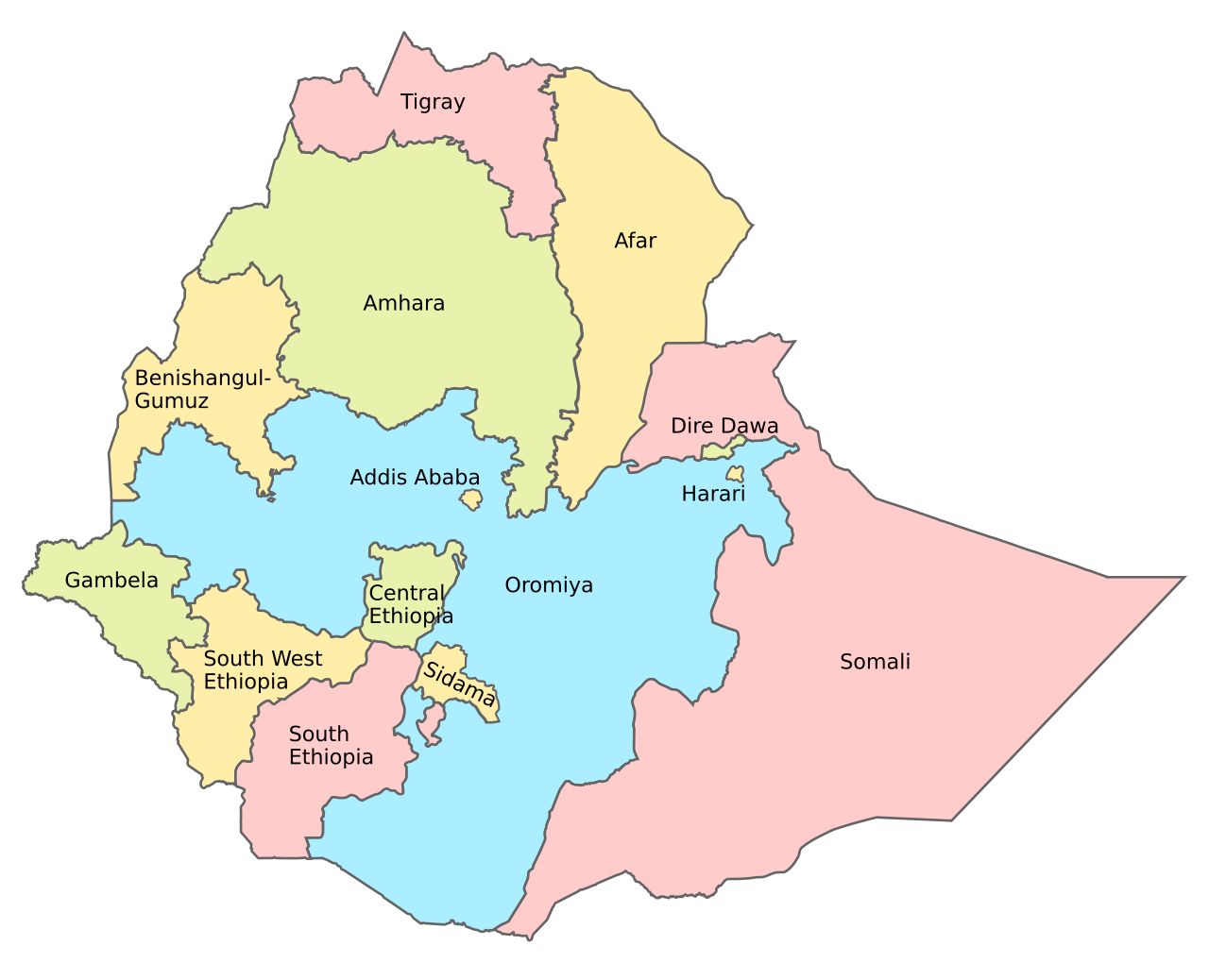


**Overview:**

Launched in 2003, as a flagship government initiative that professionalizes CHWs known as Health Extension Workers.

**Key Features:**

- Over 38,000 salaried, government-employed, mainly women, deployed in rural health posts.
- Deliver 16 packages of essential health services (disease prevention, family health, hygiene and sanitation, and health education).
- undergo one year of formal training and serve as the first contact point in the health system.
- Strong linkages with community health volunteers (Health Development Army) for outreach and mobilization.

**Impact:**

- Associated with significant improvements in immunization, sanitation, and skilled birth attendance.
- Serves as a model for task-shifting and decentralization in low-resource settings.

**Brazil**

**Family Health Strategy, (Estratégia Saúde da Família)**


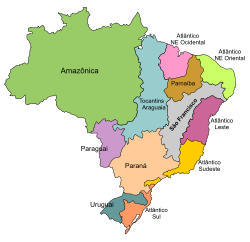


**Overview:**

launched in the 1990s, is Brazil’s principal approach to delivering comprehensive primary health care.

**Key Features:**

- Each FHS includes a physician, nurse, nurse assistant, and 4–6 CHWs (Agentes Comunitários de Saúde).
- salaried, trained, and responsible for 150–200 households, conducting home visits and linking families to care.
- Emphasis on continuity of care, health promotion, chronic disease management, and surveillance.
- Embedded in municipally managed public health system (SUS) with federal guidelines.

**Impact:**

- Linked to reductions in infant mortality, hospitalizations, and health inequities.
- Recognized globally as a successful model of integrated, community-oriented primary care.

**Supplementary Figure 4**. Selected case studies of community-based primary health care programs in Nepal, Ethiopia, and Brazil, highlighting program structure, implementation, and health system impacts, 1988–present.
