# Supplementary material for: Reimagining primary health care: a historical and contemporary scoping review of community-based primary health care models and innovations
Source: Prev Med Rep. 2026 Jan 27;62:103390. doi: 10.1016/j.pmedr.2026.103390 (PMC12877820; doi:10.1016/j.pmedr.2026.103390)
Supplement: Supplementary file 2 — Supplementary material 2 [file mmc2.docx]

**Supplementary Table 4**. Tasks traditionally performed by community health workers that may be supported or optimized through digital and artificial intelligence–enabled tools in community-based primary health care settings.

| **Task** | **Digitalisation/AI Input** | **Example** | **Time Saved (per day)** | **Reason for Savings** |
| --- | --- | --- | --- | --- |
| Health education and awareness | AI chatbots and mobile apps for health messaging | WHO’s HealthBuddy+ chatbot, mHealth apps like MomConnect | 20–30 minutes | Pre-recorded messages and chatbots reduce time spent on repetitive information |
| Disease screening and risk assessment | AI symptom checkers | Ada Health symptom checker, Babylon Health triage tool | 10–20 minutes | AI apps help streamline symptom checklists and reduce manual evaluation time |
| Monitoring treatment adherence | AI-based SMS reminders | 99 DOTS for TB adherence, Medisafe medication reminder app | 15–20 minutes | Automated SMS reminders reduce follow-up burden |
| Data collection and reporting | Digital data tools with AI validation | DHIS2 Tracker with AI-based validation, CommCare mobile data collection | 30–45 minutes | Digital forms with autofill and validation speed up data entry and reduce rework |
| Triage and referral | AI triage bots and apps | Digital triage apps like RapidPro , AI-powered Patient Triage by Infermedica | 10–15 minutes | AI decision tools provide instant referral guidance |
| Maternal and child health tracking | Mobile apps with alerts | mSakhi app for ASHAs, Safe Delivery App | 15–30 minutes | Automated scheduling and alerts reduce manual tracking |
| Mental health support | AI chatbots for mental wellbeing | Wysa AI chatbot, TalkLife peer support platform | 5–15 minutes | AI assistants manage initial support interactions |
| Chronic disease follow-up | BP tracking, reminders | Simple App for hypertension, Glucose Buddy for diabetes | 15–30 minutes | Tools like Simple App track BP, send reminders, and automate logs |
| Community engagement and mobilisation | AI messaging platforms | U-Report UNICEF, MobiliseMe AI-based outreach tool | 10–15 minutes | AI-personalised messaging replaces manual outreach |
| Health surveillance and outbreak response | Early warning via AI | ProMED-mail early warning, DHIS2 surveillance dashboards | 1–3 hours per event | Faster detection and reporting during outbreaks, saving several hours per event |
| Supply chain management | Forecasting and routing | mSupply mobile, OpenLMIS | 10–20 minutes | Automated stock level alerts avoid manual tallying and urgent ordering |
| Total Productivity Grain from Digitalisation (AI integration): Daily time saved (average): 1.5–3 hours (approximation only). Annual gain: ~78-156 hours per CHW (assuming CHW spends 1-day a week on these tasks). This equals to additional capacity to screen 156-312 patients with hypertension annually (assuming CHWs take on average 30 mins contact time per patient). | | | | |
