# Supplementary material for: Reimagining primary health care: a historical and contemporary scoping review of community-based primary health care models and innovations
Source: Prev Med Rep. 2026 Jan 27;62:103390. doi: 10.1016/j.pmedr.2026.103390 (PMC12877820; doi:10.1016/j.pmedr.2026.103390)
Supplement: Supplementary file 3 — Supplementary references [file mmc3.docx]

# Supplementary References.

S1. Srivarathan A, Jensen AN, Kristiansen M. Community-based interventions to enhance healthy aging in disadvantaged areas: perceptions of older adults and health care professionals. BMC Health Services Research. 2019;19(1):7.

S2. Rigg KK, Engelman D, Ramirez J. A Community-Based Approach to Primary Health Care. In: Arxer SL, Murphy JW, editors. Dimensions of Community-Based Projects in Health Care. Cham: Springer International Publishing; 2018. p. 105-17.

S3. Organization WH. Sparking a renaissance in mental health: strengthening community-based care for all in need. World mental health report 2022.

S4. Kupferberg N. Bringing Health Care to the Under-Served: The Mid-Level Health Practitioner in Three Countries--China, the Soviet Union, and the United States. 1990.

S5. Gizaw Z, Astale T, Kassie GM. What improves access to primary healthcare services in rural communities? A systematic review. BMC Primary Care. 2022;23(1):313.

S6. D’Apice C, Ghirotto L, Bassi MC, Artioli G, Sarli L. A realist synthesis of staff-based primary health care interventions addressing universal health coverage. Journal of global health. 2022;12:04035.

S7. Goldman L, Benjamin G, Hernández S, Kindig D, Kumanyika S, Nevarez C, et al. Advancing the health of communities and populations: a vital direction for health and health care. NAM Perspectives. 2017.

S8. Nelson A. Body and soul: The Black Panther Party and the fight against medical discrimination: U of Minnesota Press; 2011.

S9. Rössler W, Riecher-Rössler A, Meise U. Wilhelm Griesinger and the concept of community care in 19th-century Germany. Psychiatric Services. 1994;45(8):818-22.

S10. DANIŞ MZ. Community based care understanding and social services: A care model proposal from Turkey. Turkish Journal of Geriatrics. 2008;11(2).

S11. Longlett SK, Kruse JE, Wesley R. Community-oriented primary care: historical perspective. The Journal of the American Board of Family Practice. 2001;14(1):54-63.

S12. Aziato L, Omenyo CN. Initiation of traditional birth attendants and their traditional and spiritual practices during pregnancy and childbirth in Ghana. BMC Pregnancy Childbirth. 2018;18(1):64.

S13. Birhan W, Giday M, Teklehaymanot T. The contribution of traditional healers' clinics to public health care system in Addis Ababa, Ethiopia: a cross-sectional study. Journal of Ethnobiology and Ethnomedicine. 2011;7(1):39.

S14. Subedi B. Whose knowledge counts? Indigenous traditional healers and health knowledge in contemporary Nepal. Dhaulagiri Journal of Sociology and Anthropology. 2022;16:59-69.

S15. Lyngdoh JP. Contribution of Traditional Medicine Toward Primary Health Care in Meghalaya. In: Kaushik A, Suchiang A, editors. Narratives and New Voices from India: Cases of Community

S16. Subedi B. Perspective Chapter: Integrating Traditional Healers into the National Health Care System – A Review and Reflection. In: Rusangwa C, editor. Rural Health - Investment, Research and Implications. Rijeka: IntechOpen; 2023.

S17. Ijaz N, Boon H. Statutory regulation of traditional medicine practitioners and practices: the need for distinct policy making guidelines. The Journal of Alternative and Complementary Medicine. 2018;24(4):307-13.

S18. Oliver SJ. The role of traditional medicine practice in primary health care within Aboriginal Australia: a review of the literature. Journal of ethnobiology and ethnomedicine. 2013;9:1-8.

S19. Khatri RB, Endalamaw A, Erku D, Wolka E, Nigatu F, Zewdie A, Assefa Y. Enablers and barriers of community health programs for improved equity and universal coverage of primary health care services: A scoping review. BMC Primary Care. 2024;25(1):385.

S20. Assan A, Takian A, Aikins M, Akbarisari A. Challenges to achieving universal health coverage through community-based health planning and services delivery approach: a qualitative study in Ghana. BMJ Open. 2019;9(2):e024845.

S21. Long H, Zhenyu M, Duc HTT, Van MH, B. RL, S. UD, et al. Engaging village health workers in non-communicable disease (NCD) prevention and control in Vietnam: A qualitative study. Global Public Health. 2020;15(4):611-25.

S22. Olang’o CO, Nyamongo IK, Aagaard-Hansen J. Staff attrition among community health workers in home-based care programmes for people living with HIV and AIDS in western Kenya. Health policy. 2010;97(2-3):232-7.

S23. Willis-Shattuck M, Bidwell P, Thomas S, Wyness L, Blaauw D, Ditlopo P. Motivation and retention of health workers in developing countries: a systematic review. BMC health services research. 2008;8:1-8.

S24. Weber H. Age structure and political violence: a re-assessment of the “youth bulge” hypothesis. International Interactions. 2019;45(1):80-112.

S25. Jimenez EYaM, Murgai. Investing in the Youth Bulge. Finance & Development. 2006;43(3).

S26. Junaedi J, Dikrurohman D, Abdullah A. Analysis of Social Change in Rural Communities Due to the Influence of Urbanization and Globalization in Indonesia. Edunity : Kajian Ilmu Sosial dan Pendidikan. 2023;2:431-41.

S27. Friedemann ML, Buckwalter KC. Family Caregiver Role and Burden Related to Gender and Family Relationships. J Fam Nurs. 2014;20(3):313-36.

S28. Brady B, Rosenberg S, Newman C, Kaladelfos A, Kenning G, Duck-Chong E, Bennett J. Gender is dynamic for all people. Discover Psychology. 2022;2(1):41.

S29. Zhao C, Wang F, Zhou X, Jiang M, Hesketh T. Impact of parental migration on psychosocial well-being of children left behind: a qualitative study in rural China. International journal for equity in health. 2018;17(1):80.

S30. Song Q. Aging and separation from children: The health implications of adult migration for elderly parents in rural China. Demogr Res. 2017;37:1761-92.

S31. Moen P, Robison J, Fields V. Women's work and caregiving roles: a life course approach. J Gerontol. 1994;49(4):S176-86.S32 (Original Ref #109): Dahal P et al. Gender inequality in Nepal. 2022.

S33. Jat AS, Grønli T-M, editors. Harnessing the digital revolution: a comprehensive review of mHealth applications for remote monitoring in transforming healthcare delivery. International Conference on Mobile Web and Intelligent Information Systems; 2023: Springer.

S34. Blocker A, Datay MI, Mwangama J, Malila B. Development of a telemedicine virtual clinic system for remote, rural, and underserved areas using user-centered design methods. DIGITAL HEALTH. 2024;10:20552076241256752.

S35. Kumar Y, Gupta S, Singla R, Hu YC. A Systematic Review of Artificial Intelligence Techniques in Cancer Prediction and Diagnosis. Arch Comput Methods Eng. 2022;29(4):2043-70.

S36. Wu M, Du X, Gu R, Wei J. Artificial intelligence for clinical decision support in sepsis. Frontiers in Medicine. 2021;8:665464.

S37. Dhamani N. Introduction to generative AI: Simon and Schuster; 2024.

S38. Larasati R. Inclusivity of AI Speech in Healthcare: A Decade Look Back. arXiv preprint arXiv:250510596. 2025.

S39. Klingbeil A, Grützner C, Schreck P. Trust and reliance on AI—An experimental study on the extent and costs of overreliance on AI. Computers in Human Behavior. 2024;160:108352.

S40. Mondal H, Mondal S. Chapter Thirteen - Ethical and social issues related to AI in healthcare. In: Srivastava A, Mishra V, editors. Methods in Microbiology. 55: Academic Press; 2024. p. 247-81.
